# The Resilience Journal: Exploring the Potential of Journal Interventions to Promote Resilience in University Students

**DOI:** 10.3389/fpsyg.2021.702683

**Published:** 2021-10-06

**Authors:** Max S. Lohner, Carmela Aprea

**Affiliations:** Chair of Economic and Business Education – Instructional Systems Design and Evaluation, Business School, University of Mannheim, Mannheim, Germany

**Keywords:** resilience, university students, journal intervention, online intervention, broaden-and-build theory, well-being, higher education, COVID-19

## Abstract

Given the prevalence of mental health issues among university students, they must be regarded as a vulnerable population. Resilience interventions offer one potential means of strengthening students’ capacity to overcome academic challenges and external threats. This is all the more urgent in light of the additional difficulties caused by the current COVID-19 pandemic, such as the demands of remote learning. The present study is a first step toward designing and evaluating an appropriate dynamic resilience intervention for students. The design of the *Resilience Journal* intervention draws on insights from expressive writing and positive writing research and focuses on reflection on daily challenges. In this online intervention, 100 business school students (66% female, M_*age*_ = 23.74) at a German university were randomly assigned to two groups and completed two different versions of the *Resilience Journal* for 5 days. The two versions focused, respectively on broadening attention to challenges and priming attention to mastered challenges. In a pre-post design, two resilience measures and one measure of life satisfaction were used to assess intervention outcomes. Additionally, a newly developed rating scale was used for daily monitoring of dynamic resilience. While both groups showed a significant increase in resilience as measured by the Brief Resilience Scale, that increase could not be attributed directly to the intervention, as there were no group differences, and the design did not include a control group. The other resilience and life satisfaction measures showed no significant change. This first implementation confirms the potential of the *Resilience Journal* and indicates directions for the development of dynamic resilience interventions and measures in future studies. To further study the potential of such a positive psychology intervention, future research necessitates the inclusion of control groups.

## Introduction

Despite the traditional positive view of entering university, many studies have shown that this stage of life poses multiple challenges for students that may increase psychological disturbance (e.g., Fisher and Hood, 1987; Leary and DeRosier, 2012; Hussain et al., 2013). In line with those findings, recent research in Germany revealed that more than 80% of higher education students report time- and performance-related pressures (Herbst et al., 2016). About 25% reported symptoms of burnout (Grützmacher et al., 2018) while 17.4% said they experienced anxiety, and 15.6% exhibited symptoms of depression (Grützmacher et al., 2018). Compared to their non-university peers, higher education students are more often diagnosed with depressive episodes and affective or anxiety disorders (Techniker Krankenkasse, 2015), as well as higher stress levels than those of working adults in general (Herbst et al., 2016). While mental health problems were already known to be more prevalent among higher education students before the pandemic, there is emerging evidence that the pandemic has created additional risks to their well-being (e.g., Essadek and Rabeyron, 2020; Liu et al., 2020). To that extent, students should be considered a vulnerable population, and ensuring their well-being seems an important organizational goal for universities. However, this creates a dilemma, as universities have a responsibility to challenge students to develop the necessary career competencies, and those challenges cannot simply be reduced to manage the potential impact on well-being.

One possible way of mitigating this dilemma would be to find ways of enhancing students’ resilience to enable them to cope with the new and challenging organizational environment they encounter in the university and so maintain their well-being despite facing challenges (e.g., Pidgeon and Keye, 2014; Turner et al., 2017). The building of resilience is based on the neuronal plasticity of the brain; the ability of the brain to be shaped by experiences (Nelson, 1999; Masten, 2001; Curtis and Cicchetti, 2003; Lerner et al., 2012). While this neuronal plasticity can have negative effects on the brain, when confronted with stressful experiences, it also provides the ability to adapt to changes and learn from experiences (e.g., Nelson, 1999; Curtis and Cicchetti, 2003). The ubiquity of relative plasticity across the life span suggests that individuals can adapt successfully and be resilient even later in their lives (e.g., Nelson, 1999; Lerner et al., 2012). According to Tabibnia and Radecki (2018) cognitive and behavioral pathways can influence the neuroplasticity and boost resilience. Despite this important bio-psychological foundations of resilience, other factors such as current experiences, social context, timing of adverse evet(s), and experiences, as well as the developmental history of the individual influence resilience (Curtis and Cicchetti, 2003). Resilience can therefore be seen as a complex multidimensional construct (Luthar et al., 2000). During the transition to university especially the external influences on individual’s resilience change. In this phase of live family support often decreases and additional demands for autonomy, self-regulation and academic pressure require adaption (e.g., Fisher and Hood, 1987; Bitsika et al., 2010; Leary and DeRosier, 2012; Houston et al., 2017). Supporting the resilience of university students is particularly important in this phase of life. According to Archana and Singh (2014, 228), “resilience has emerged as one of the most important factors that contribute towards the well-being of students.” Existing research suggests that appropriate interventions can increase resilience and well-being among higher education students (e.g., Galante et al., 2018; Hill et al., 2018), reducing stress, anxiety, and depression (e.g., Steinhardt and Dolbier, 2008; Houston et al., 2017; Akeman et al., 2020). Resilience is linked to greater life satisfaction and academic progress, especially for vulnerable students (van Breda, 2018).

Despite the observed positive effects of resilience and resilience interventions, relevant research insights remain limited. Most definitions of resilience refer to the two core concepts of *adversity* and *positive adaptation* (Fletcher and Sarkar, 2013), but there is no gold standard how resilience should be defined or measured (Windle et al., 2011; Calitz, 2018), making it difficult to operationalize resilience and compare scientific results or apply them in practice. This diversity of definitions reflects both the complex multidimensional nature of the construct and the historical development of how resilience is understood (Luthar et al., 2000). While pioneering researchers defined resilience as a stable lifelong trait (e.g., Werner, 1993; Block and Kremen, 1996), subsequent approaches viewed it as a “dynamic process encompassing positive adaptation within the context of significant adversity” (Luthar et al., 2000, 543)–in other words, resilience came to be seen as a changing process rather than a stable trait (e.g., Luthar et al., 2000; Masten, 2001). Today, researchers continue to pursue both of these approaches and multiple definitions are used (e.g., Robertson et al., 2015; Chmitorz et al., 2018a; Joyce et al., 2018; Brewer et al., 2019; Linz et al., 2020). In the field of training and teaching, resilience is more often characterized as a dynamic process (Linz et al., 2020), and the present study adopts this recommendation and the definition proposed by Luthar et al. (2000).

Given these differing definitions, it is unsurprising that intervention researchers employ different scales to measure resilience (e.g., Robertson et al., 2015; Chmitorz et al., 2018a; Joyce et al., 2018; Brewer et al., 2019; Linz et al., 2020). Importantly, these different scales do not measure exactly the same construct (Joyce et al., 2018), and their results must therefore be treated with caution. In particular, the more commonly used scales are not ideal for measuring dynamic resilience processes over shorter time intervals (e.g., daily). In addition, the social distancing regulations associated with the COVID-19 pandemic have created a pressing need for effective online interventions, which remain rare in this context (e.g., Robertson et al., 2015; Joyce et al., 2018; Brewer et al., 2019; Linz et al., 2020).

In light of the known positive outcomes of resilience interventions for higher education students and the existing limitations of resilience research, the present study represents the first step in a larger research project, which aims at understanding, measuring, and fostering resilience as a dynamic process. The goal of this initial study was to assess the potential of a novel online intervention to support university students’ resilience and well-being during distance learning. To develop this intervention, we adapted findings from the existing research on writing interventions. Expressive writing interventions are traditionally used to help participants to recover from traumatic events by promoting disclosure, habituation, and desensitization (Wing et al., 2006; Burton and King, 2009). According to Glass et al. (2019), such interventions are also a very effective means of promoting resilience development.

Beyond the domain of trauma, the growing body of positive psychology interventions include positive writing interventions (Reiter and Wilz, 2016). Although they developed from expressive writing (Wing et al., 2006), positive writing interventions focus on remembering and reflecting on positive experiences and associated positive emotions (Reiter and Wilz, 2016). In particular, diary and journal interventions such as the *Gratitude Journal* (Emmons and McCullough, 2003), where participants regularly record five experiences for which they are grateful, have been widely evaluated. In general, the *Gratitude Journal* is reported to enhance life appraisal and positive affect while reducing negative affect (e.g., Emmons and McCullough, 2003). Among college students this approach has been shown to increase gratitude, life satisfaction, and university adaptation (Işık and Ergüner-Tekinalp, 2017).

Other positive psychology interventions have also confirmed the promising effects of positive writing. For example, Cohn et al. (2009) reported increased resilience among higher education students who reported positive emotions for 28 days. In an evaluation of the *Three Good Things in Life* intervention, which asks participants to write down three things that went well each day, Seligman et al. (2005) showed that this had the strongest long-term effects among different happiness interventions. In a related study, Risch and Wilz (2013) asked participants to write for 4 weeks in a *Resource Journal* about their interpersonal and intrapersonal resources, and this was found to have positive effects on mood and emotion regulation.

These insights highlight the potential of positive writing interventions in non-traumatic contexts. To the best of our knowledge, however, there is as yet no published account of an intervention based on journaling of resilience experiences. Given the reported effects of expressive writing (Glass et al., 2019) and positive writing (Cohn et al., 2009) on resilience, it seems worthwhile to adapt this approach for resilience enhancement. Following Tabibnia and Radecki (2018) journaling can influence the neuroplasticity and resilience *via* the cognitive (i.e., emotion disclosure) and the behavioral pathway (i.e., gratitude). In addition, this method lends itself to online delivery, which has become a critical issue during the COVID-19 pandemic. Both Cohn et al. (2009) and Seligman et al. (2005) delivered their interventions online, and a more recent publication by Kern et al. (2018) reported that a number of web- and app-based positive psychology interventions have employed the journaling method, indicating its suitability for online distance learning delivery.

Despite empirical evidence of the effectiveness of positive writing, the reasons for this remain unclear (Reiter and Wilz, 2016). Some authors have proposed an explanation based on broaden-and-build-theory (e.g., Emmons and McCullough, 2003; Burton and King, 2009). The primary claim of this theory is the *broadening* effect (Conway et al., 2012) of positive emotions on attention and cognition (Fredrickson, 2001). Broadened attention incorporates experiences from one’s surroundings that would otherwise have been excluded, and broadened cognition expands one’s thinking, cognitive flexibility, and creativity (Conway et al., 2012). According to this theory, broadening attention and cognition triggers momentary thought-action repertoires that can weaken or transform negative emotions to provide resources for coping with adversities.

Based on this theory, Burton and King (2009) proposed that “writing about a positive experience is, itself, a positive experience” (868) that can broaden cognition. In this regard, Fredrickson (2001) theorized that positive emotions foster a positive upward spiral over time, resulting in increased resilience and well-being. Cohn et al.’s (2009) findings support this claim and show that the relationship between positive emotions and life satisfaction (as an indicator of well-being) is fully mediated by the change in resilience. The theoretical assumptions and empirical evidence underpinning broaden-and-build-theory support the view that journaling resilience experiences is likely to increase resilience.

Other authors have proposed underlying mechanisms beyond broaden-and-build-theory. Rather than a general broadening of attention, these explanations suggest that writing interventions direct attention in particular ways. For example, Risch and Wilz (2013) explained the effectiveness of their positive writing intervention in terms of resource priming, and Wing et al. (2006) described positive writing as an opportunity for the writer to gain a sense of mastery. In other words, the priming of resources and abilities may result in their more frequent use, so increasing resilience and well-being.

As mentioned earlier, resilience is typically explained in terms of two core concepts, *adversity* and *positive adaptation* (Fletcher and Sarkar, 2013), which underpin two distinct explanations of the effectiveness of positive writing interventions. While directing attention to resources and mastery emphasizes *positive adaptation* and ways of achieving it, broaden-and-build-theory posits a general broadening of attention and cognition that involves both core concepts. It can be hypothesized that these alternative approaches vary in their effectiveness because they address adversities differently. A design that focuses on adversities may have negative effects if it primes negative emotions, but an intervention that emphasizes disclosure, habituation, and desensitization to adversities may have positive effects (e.g., Wing et al., 2006; Burton and King, 2009).

In sum, journal interventions are a widely used and potentially effective means of increasing resilience. To our knowledge, however, the existing literature does not include an online journal intervention that specifically addresses resilience. The *Resilience Journal* described in this explorative study is grounded in theory and was tested empirically in the vulnerable population of university students. In contrast to existing journal-based studies of resilience (e.g., Cohn et al., 2009), we asked university students to reflect on their resilience-related experiences in a daily writing intervention to explore the influences of this activity. In an attempt to clarify the underlying mechanisms of positive writing, two separate versions of the journal were implemented. While the *Attention Version* adopts broaden-and-build-theory and focuses on a general broadening of attention, and the *Mastery Version* primes attention to resources and abilities. The respective effects on student resilience and well-being were evaluated in terms of the following hypotheses:

**H1:** University students who complete a daily resilience journal develop greater resilience and well-being over time.

**H2:** Interventions based on the *Attention Version* and the *Mastery Version* differ in their effects on student resilience and well-being.

## Materials and Methods

### Participants and Recruitment

Students of economic and business education at the University of Mannheim were contacted by email during the 1st week of August 2020. Based on the information provided, 111 students registered and gave informed consent for data collection. Of these, 103 students completed the pre- and post-test. Three students who completed less than half of the daily journals were excluded from the subsequent analysis on grounds of insufficient exposure to the intervention. The final sample included 100 university students between the ages of 19 and 30 years (*M* = 23.74, *SD* = 2.44) who were majoring in economic and business education. The participants had completed between 1 and 14 semesters (*M* = 5.68, *SD* = 3.32, 47% bachelor, and 53% master), and the gender distribution of 34% males and 66% females was representative of the study program as a whole. Participation was voluntary, but optional course credits could be awarded for participation.

### Intervention

To create a journal intervention, we formulated a daily task that involved reflection on daily challenges. Following Emmons and McCullough (2003) and Seligman et al. (2005), the formulation of the *Resilience Journal* drew on insights from the *Gratitude Journal* and the *Three Good Things in Life* interventions. To explore the mechanisms underlying the intervention, two journal versions were formulated.

Based on broaden-and-build-theory, the *Attention Version* was designed to broaden attention to challenging daily experiences (Conway et al., 2012), recording both successes and failures in overcoming those challenges. Theoretical considerations informed the following instruction to participants.

*Every day, we face many challenges, both small and big, in private and academic contexts. Think back over the past day and enter three challenges that you encountered in the field below. For each challenge, write down what specifically was challenging for you.*

The *Mastery Version* was designed to activate the posited mechanisms of resource priming (Risch and Wilz, 2013) and mastery (Wing et al., 2006). To that end, this version directed the participant’s attention to challenges that were successfully mastered, based on the following instruction.

*Every day, we master many challenges, both small and big, in private and academic contexts. Think back over the past day and enter three challenges that you mastered today in the field below. For each challenge, write down how you mastered it.*

For the purposes of comparison, participants were randomly assigned to one of the two versions, which they completed each day for 5 days.

### Measures

#### Brief Resilience Scale

Originally developed by Smith et al. (2008), the Brief Resilience Scale (BRS) is a six-item scale that measures resilience as the “ability to bounce back” (195), based on items such as “I tend to bounce back quickly after hard times” rated on a 5-point Likert scale (1 = *strongly disagree* to 5 = *strongly agree*). This scale has been recommended for use in resilience research for its validity (Linz et al., 2020). For present purposes, we used the German version (Chmitorz et al., 2018b), which achieved good reliability (Cronbach’s alpha 0.85) for a German sample.

#### Connor-Davidson Resilience Scale

The Connor-Davidson Resilience Scale (CD-RISC) (Connor and Davidson, 2003) comprises 25 items (e.g., ability to adapt to change) measuring resilience on a 5-point Likert scale (0 = *rarely true* to 4 = *true nearly all of the time*). This is the most widely used scale for measuring resilience (e.g., Salisu and Hashim, 2017). For present purposes, we used the German version, which has achieved a high Cronbach’s alpha of 0.90 (Sarubin et al., 2015).

#### Satisfaction With Life Scale

The Satisfaction with Life Scale (SWLS) (Diener et al., 1985) is a self-report assessment of global life satisfaction as an element of subjective well-being. The scale includes five items (e.g., “I am satisfied with my life”) rated on a 7-point Likert scale (1 = *strongly disagree* to 7 = *strongly agree*). The German version of the SWLS shows very good internal consistency (Cronbach’s alpha 0.92) (Glaesmer et al., 2011).

#### Monitoring of Actual Resilience State

To account for pre-post differences and to evaluate the daily effects of the two journal versions, a third resilience measure was included to detect dynamic changes in resilience from day to day. As no existing resilience measure was appropriate for daily use, we developed a new scale. The *Monitoring of Actual Resilience State* (MARS) scale includes eight items (see Table 1) rated on a slider control scale (1 = *strongly disagree* to 100 = *strongly agree*).

**TABLE 1:** Items related to Monitoring of Actual Resilience State (MARS).

**MARS Items**
Today…
(1) I had support when I needed it.
(2) I could rely on myself to overcome challenges.
(3) I did not give up in the face of adversities.
(4) I dealt well with negative emotions.
(5) My actions did not lead to a higher goal.^1^
(6) I had difficulties with recovering from stress.^1^
(7) I should have tried harder to achieve my goals.^1^
(8) I lacked something to overcome challenges.^1^

*^1^reverse items.*

#### Short 10-Item Version of the Big Five Inventory

The short 10-item version of the Big Five Inventory (BFI-10) (Rammstedt and John, 2007) was included as a control measure. BFI-10 measures the Big Five personality traits (extraversion, agreeableness, conscientiousness, neuroticism, and openness); each trait is measured on two items, rated on a 5-point Likert scale (1 = *disagree strongly* to 5 = *agree strongly*). Again, we used the German version, which was also published by Rammstedt and John (2007).

### Procedure

A pre-post design was chosen to evaluate the two versions of the *Resilience Journal*. After registering and giving their informed consent, participants completed the pre-test, which included the BRS, CD-RISC, SWLS, and BFI-10, as well as demographic items (gender, age, study semester, and study program). Using a random number generator, we then assigned participants randomly to one of two groups; Group 1 (*n* = 50, *M*_*Age*_ = 23.90, *SD*_*Age*_ = 2.42, and 36% male) were asked to complete the *Attention Version* of the *Resilience Journal* for 5 days while Group 2 (*n* = 50, *M*_*Age*_ = 23.58, *SD*_*Age*_ = 2.47, and 32% male) completed the *Mastery Version* of the *Resilience Journal* for the same period. In addition, all participants were asked to complete the MARS scale each day. After 5 days, participants completed the post-test, which measured the same items as the pre-test. At each measurement point, each participant was identified by their individual code, enabling evaluation of individual changes while preserving anonymity.

All measurement scales and the assigned version of the *Resilience Journal* were completed online through the SoSci-Survey program; participants could use any device with internet access. The intervention took place during the COVID-19 pandemic (August 10–14, 2020); pre- and post-tests could be completed up to 3 days before and after the intervention, respectively.

### Data Analyses

Mean scores and standard deviations were calculated for all variables as the basis for further analyses. Pre-requirements for hypothesis testing were evaluated, and reliability and correlations were calculated for all dependent variables to assess measurement quality. To assess the effects of the two versions of the *Resilience Journal* intervention, we compared the results of the BRS, CD-RISC, and SWLS as between-group factors in a one-way MANOVA. To test Hypothesis 1, we performed MANOVA and *post hoc* ANOVAs of time effects. To test Hypothesis 2, we examined time x group interactions in the same MANOVA, followed by *post hoc* ANOVAs and discriminant analysis. Additional ANOVAs were computed to assess changes in MARS. The multilevel reliability of MARS was analyzed using Mplus Version 8.6; all other analyses were performed using IBM SPSS Statistics Version 27.0.0.0. A significance level of α = 0.05 was used throughout. For multiple testing, the Bonferroni correction was applied.

## Results

### Pre-requirements

To establish pre-requirements for statistical procedures, we tested for pre-existing group differences and violations of homogeneity of variance and normal distribution. A one-way MANOVA, including all pre-test variables, found no significant group differences (*F*(13,83) = 0.440, *p* = 0.950). Further exploration of the pre-test measures and Day 1 MARS data found no significant differences in between-group t-tests and no significant violation of homogeneity of variance in Levene tests (see Supplementary Table 1). No variable exceeded the acceptable skewness of ±2, and only the demographic variables study semester and study program violated kurtosis of ±2 (see Supplementary Table 1). Normal distribution of all other variables was accepted.

Measurement scale reliability and validity were assessed by calculating Cronbach’s alpha and correlations for all dependent pre-post-variables. BRS, CD-RISC, and SWLS achieved acceptable Cronbach’s alpha values (α > 0.70; see Table 2). All subscales of BFI-10 other than extraversion showed a Cronbach’s alpha of less than 0.70. Cronbach’s alpha values for the daily MARS ranged from α = 0.64 to α = 0.81; only Day 3 values fell below 0.70 (see Table 2). To take account of the multi-level structure of MARS, an intraclass correlation (ICC) and multi-level reliability were computed. ICC showed a relatively low value of 0.29. Multi-level reliability estimation using CFA as proposed by Geldhof et al. (2014) returned a total reliability of α = 0.75, with between-person reliability of α = 0.79 and within-person reliability of α = 0.73.

**TABLE 2:** Pre-post variables: correlations and Cronbach’s alpha.

**Variable**		**1a**	**1b**	**2a**	**2b**	**3a**	**3b**	**4c**	**4d**	**4e**	**4f**	**4g**	**5a**	**5b**	**6a**	**6b**	**7a**	**7b**	**8a**	**8b**	**9a**	**9b**	**α**
(1) BRS	(a) Pre	−																					0.80
	(b) Post	0.70	−																				0.79
(2) CD-RISC	(a) Pre	0.49	0.42	−																			0.83
	(b) Post	0.46	0.40	0.80	−																		0.84
(3) SWLS	(a) Pre	0.31	0.28	0.33	0.23	−																	0.86
	(b) Post	0.31	0.36	0.38	0.35	0.83	−																0.85
(4) MARS	(c) Day 1	0.17	0.09	0.22	0.13	0.39	0.39	−															0.73
	(d) Day 2	0.27	0.27	0.22	0.33	0.19	0.22	0.31	−														0.81
	(e) Day 3	0.18	0.12	0.27	0.26	0.20	0.30	0.26	0.26	−													0.64
	(f) Day 4	0.27	0.25	0.08	0.19	0.27	0.34	0.05	0.25	0.40	−												0.78
	(g) Day 5	0.17	0.32	0.02	0.20	0.24	0.40	0.31	0.28	0.41	0.42	−											0.75
(5) BFI-10 N	(a) Pre	–0.41	–0.40	–0.35	–0.30	−0.15	−0.18	−0.16	−0.17	−0.14	–0.23	−0.15	−										0.57
	(b) Post	–0.49	–0.46	–0.45	–0.57	−0.15	−0.19	−0.06	–0.22	−0.19	–0.29	–0.27	0.64	−									0.50
(6) BFI-10 C	(a) Pre	−0.06	0.01	0.07	−0.03	0.20	0.18	0.29	0.12	0.09	0.11	0.07	−0.08	0.08	−								0.50
	(b) Post	0.06	0.10	0.15	0.18	0.19	0.27	0.21	0.23	0.25	0.21	0.17	−0.04	−0.09	0.73	−							0.39
(7) BFI-10 E	(a) Pre	0.07	0.08	0.06	0.15	0.14	0.14	0.11	0.13	−0.03	0.05	0.06	−0.01	–0.21	0.00	0.09	−						0.77
	(b) Post	0.13	0.19	0.10	0.26	0.09	0.16	0.05	0.12	−0.03	0.10	0.18	−0.17	–0.40	−0.08	0.03	0.87	−					0.85
(8) BFI-10 O	(a) Pre	−0.05	0.02	0.15	0.19	0.16	0.12	0.10	0.19	0.06	0.08	0.09	−0.02	−0.03	0.05	0.09	0.05	0.07	−				0.65
	(b) Post	0.05	0.04	0.23	0.24	0.17	0.14	0.04	0.07	0.15	0.06	0.09	−0.04	−0.14	0.06	0.17	0.14	0.15	0.78	−			0.68
(9) BFI-10 A	(a) Pre	0.04	0.00	0.10	0.09	0.09	0.01	0.19	0.10	−0.06	−0.03	0.13	0.01	0.03	0.09	0.01	0.00	−0.04	0.10	0.05	−		0.17
	(b) Post	0.03	−0.04	0.02	0.07	0.10	0.09	0.21	0.05	−0.02	0.07	0.18	−0.01	0.03	0.08	−0.02	−0.01	−0.04	−0.04	−0.08	0.81	−	0.22

*Significant correlations (p < 0.05) in black print; (1) BRS, brief resilience scale; (2) CD-RISC, connor-davidson resilience scale; (3) SWLS, satisfaction with life scale; (4) MARS, monitoring of actual resilience state; (5) BFI-10 N, big five inventory 10 – neuroticism; (6) BFI-10 C, big five inventory 10 – conscientiousness; (7) BFI-10 E, big five inventory 10 – extraversion; (8) BFI-10 O, big five inventory 10 – openness; (9) BFI-10 A, big five inventory 10 – agreeableness; (a) Pre, pre-test variable; (b) Post, post-test variable; (c) Day 1, first day of intervention; (d) Day 2, second day of intervention; (e) Day 3, third day of intervention; (f) Day 4, fourth day of intervention; (g) Day 5, fifth day of intervention; and α, Cronbach’s alpha.*

All resilience measures (BRS, CD-RISC, and MARS) showed medium-to-low correlations to each other. Correlations of the resilience measures to life satisfaction were positive, and to neuroticism they were negative; both in a medium to low magnitude (Table 2).

### Hypothesis 1: Effectiveness of the Resilience Journal

The results indicate the significant impact of time point (*F*(1,98) = 6.48, *p* = 0.012, and η^2^ = 0.06) for both groups combined. In *post hoc* analyses, separate one-way ANOVAs were computed for every variable included in the MANOVA (see Table 3). BRS results for both groups combined revealed a significant increase over time in resilience (*F*(1,98) = 9.91, *p* = 0.002, and η^2^ = 0.092). There were no mean differences in CD-RISC results, and a *post hoc* one-way ANOVA revealed no significant time effects (*F*(1,98) = 0.089, *p* = 0.766, and η^2^ = 0.001). Mean SWLS values increased slightly from pre- to post-test, but the *post hoc* ANOVA showed that this increase was not significant (*F*(1,98) = 1.17, *p* = 0.282, and η^2^ = 0.012). In the case of MARS, a one-way ANOVA found no significant effect of time (*F*(4,86) = 1.23, *p* = 0.31, and η^2^ = 0.054) at any point.

**TABLE 3:** Results of pre-post measures: means, standard deviations, and ANOVA.

**Variable**	**Pre**			**Post**			**ANOVA**
	**MV**	**AV**	**Total**	**MV**	**AV**	**Total**	
BRS	3.19 (0.76)	3.36 (0.60)	3.27 (0.68)	3.45 (0.75)	3.42 (0.59)	3.44 (0.67)	Time: *F* = 9.91**, η^2^ = 0.092 Interaction: *F* = 2.14, η^2^ = 0.037
CD-RISC	2.89 (0.37)	2.82 (0.39)	2.86 (0.38)	2.86 (0.38)	2.87 (0.38)	2.86 (0.38)	Time: *F* = 0.89, η^2^ = 0.001 Interaction: *F* = 3.74, η^2^ = 0.021
SWLS	5.33 (1.11)	5.37 (0.85)	5.35 (0.98)	5.38 (1.01)	5.44 (0.90)	5.41 (0.96)	Time: *F* = 1.17, η^2^ = 0.012 Interaction: *F* = 0.0674, η^2^ = 0.001

Standard deviations in parentheses; MV, mastery version of the resilience journal; AV, attention version of resilience journal; Total, both conditions combined; ANOVA, ANOVA results; BRS, brief resilience scale; CD-RISC, connor-davidson resilience scale; SWLS, satisfaction with life scale. **p > 0.01.

### Hypothesis 2: Effectiveness of Journal Versions

**FIGURE 1:**
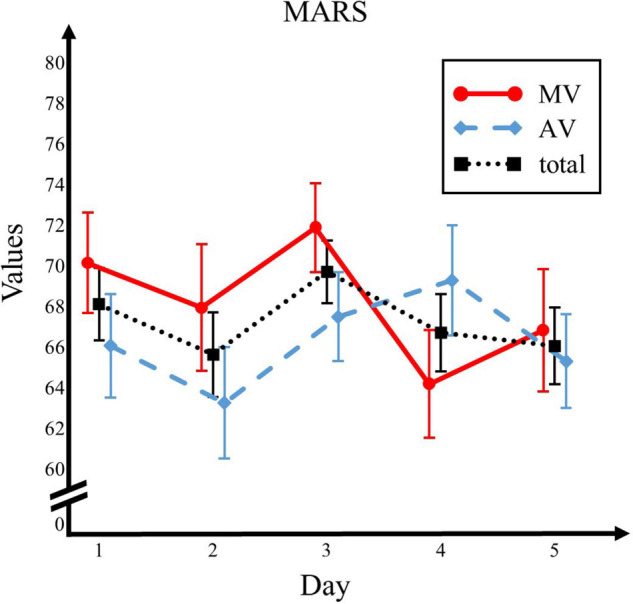
Trends in Monitoring of Actual Resilience State (MARS). MV, mastery version of resilience journal; AV, attention version of resilience journal; Total, both conditions combined.

Interactions in MANOVA and *post hoc* ANOVAs were analyzed to identify between-group differences. A one-way MANOVA found no significant time x group interaction (*F*(1,98) = 0.32, *p* = 0.574, and η^2^ = 0.003). In the individual *post hoc* analyses, one-way ANOVAs established that BRS time x group interaction was not significant (*F*(1,98) = 3.74, *p* = 0.056, and η^2^ = 0.037). *Post hoc* one-way ANOVAs also found no significant time × group interaction for the CD-RISC (*F*(1,98) = 2.14, *p* = 0.147, and η^2^ = 0.021) and SWLS (*F*(1,98) = 0.06, *p* = 0.808, and η^2^ = 0.001). For all three variables, *post hoc* discriminant analysis revealed no significant discriminant function (Λ = 0.94, χ^2^ = 5.43, *df* = 9, and *p* = 0.80). A one-way ANOVA also showed no significant effect of time x group interaction for MARS (*F*(4,86) = 1.52, *p* = 0.20, and η^2^ = 0.066) (see Figure 1).

## Discussion

This explorative study represents a first step toward the design and evaluation of an online journal-based intervention addressing daily challenges and resilience experiences. The journaling approach was chosen for its known effectiveness in positive psychology and trauma research, and for its suitability for online delivery. To explore the mechanisms that determine the effectiveness of such interventions, we compared two distinct versions of the journal and accompanying instructions. Unlike existing journal-based studies of resilience, we included a daily writing intervention to explore the influences of university students’ reflections on their resilience-related experiences.

In relation to Hypothesis 1, the pre-post MANOVA and ANOVA results revealed a significant increase in resilience as measured by the BRS, but there was no significant increase in the other resilience measures (CD-RISC, MARS). Despite an increase in mean values for life satisfaction, there were no significant effects. In relation to Hypothesis 2, there were no significant differences on any variable between the two versions of the *Resilience Journal*.

Following Ellis (2010), the effect sizes found here can be interpreted as follows. The effects of time on resilience (H1) as measured by BRS were medium and small on SWLS. Only BRS showed a significant increase, although mean SWLS differences were positive in direction. As measured by CD-RISC, there was no time effect for resilience. For time x group interaction (H2), small effects were found for resilience as measured by BRS and CD-RISC. However, these effects were not significant in either case and differed in direction. Mean differences in BRS scores indicate a stronger increase in resilience among those using the *Mastery Version*. Mean CD-RISC scores show a small decrease in resilience among those using the *Mastery Version* while those using the *Attention Version* show a small increase. SWLS results show no effect of time x group interaction on this variable. Although most of the results fell short of significance, the reported effect sizes align with the medium to small effect sizes reported in other resilience and positive psychology interventions in similar contexts (e.g., Davis et al., 2016; Houston et al., 2017; Hill et al., 2018; Akeman et al., 2020; Armenta et al., 2020; Linz et al., 2020).

As measured by BRS, resilience increased significantly from pre- to post-test, but the other resilience measures indicate no such effect. The observed differences and medium-to-low correlations between resilience measures align with Joyce et al.’s (2018) view that the various scales are not measuring the same construct. Despite the increase in BRS values, H1 is not fully supported, as the observed increase refers only to resilience as the ability to bounce back (Smith et al., 2008). The CD-RISC literature reports that the scale measures trait resilience (e.g., Singh and Yu, 2010; Wollny and Jacobs, 2021), which should not change easily, and the absence of any effect on this scale aligns with this theoretical view. The differences in resilience measures highlight that operationalization of resilience solely *via* these scales could be problematic.

The MARS resilience measure was used for the first time in this study, and the findings reflect its non-validated status. According to Nezlek (2017), diary measures are often reported as less reliable than classic trait measures, and evaluation standards should be more relaxed. Nevertheless, MARS was found to offer good reliability at daily level, as all but one measure of internal consistency exceeded a Cronbach’s alpha value of 0.70. Multi-level reliability estimation using CFA aligned with this observation, with alpha values above 0.70 for the separate levels and the overall model. The results indicate that between-person reliability was slightly better than within-person (see Geldhof et al., 2014), aligning with low-to-medium re-test correlations and ICC values indicating that only 29% of the variance can be attributed to stable personal attributes. This suggests that the MARS instrument is sufficiently reliable and dynamic to account for daily variations. In relation to construct validity, MARS was correlated with BRS and CD-RISC at a medium-to-low level. This suggests that MARS is similar in some respects but not identical to those more established measures of resilience or associated constructs. Correlations with satisfaction with life and the personality traits of conscientiousness and neuroticism align with earlier research on resilience among university students (e.g., Wilson et al., 2019). This suggests that MARS is useful as a measure of daily dynamic resilience, but further research is needed to clarify how dynamic resilience relates to other operationalizations. All MARS correlations were medium to low; the other scales used here were not based on a dynamic understanding, and the Big Five and CD-RISC are trait measures, which may account for the low-to-medium correlations. The non-significant MARS results call into question whether the scale lacks the necessary construct validity to detect dynamic variations in resilience or whether dynamic resilience did not change significantly within the given timeframe. The non-significant results are also contrary to the significant change detected by BRS. These differences could indicate a lack of convergent validity. In short, further validation is needed to determine whether MARS can adequately detect changes in dynamic resilience and behaves like other resilience scales. Linz et al. (2020) also recommends including biological resilience measures more often in research studies. The relation of neuronal plasticity and resilience provides a biological basis for measuring resilience *via* physiological measures (e.g., Curtis and Cicchetti, 2003). Further evaluation of the validity of MARS and estimating if the time of exposure to the intervention had physiological effects could have provided additional objective insights.

Regarding the non-significant increase in satisfaction with life, it remains unclear whether the intervention had no effect on this variable or whether the interval between pre- and post-test was too short to reveal any such effect. According to Cohn et al. (2009), resilience can contribute to greater life satisfaction, but no such effect was observed here beyond the increase measured by BRS. The absence of a control group prevents attribution of any changes in resilience or life satisfaction solely to the intervention, and other factors may have contributed.

In relation to H2, the results cannot confirm the superiority of either version of the *Resilience Journal*. It is therefore impossible to clarify the underlying mechanism or whether broadening attention is more effective than directing the focus to resources and abilities. As the broaden-and-build theory suggests that directing attention to mastered experiences may also foster positive emotions (e.g., Burton and King, 2009), the two versions of the *Resilience Journal* may offer two distinct routes to the same mechanism. This first exploration of the two journal versions did not control for positive emotions and broadening of attention, and future studies should do so to clarify the mechanisms at work in journal interventions.

### Strengths and Limitations

This study represents the first implementation of two different versions of the *Resilience Journal*. The aim of this study was to explore the potential of journal interventions to increase resilience in students and create a starting point for further studies. Due to its exploratory nature and the development of a dynamic longitudinal intervention and measure, this study shows some strength, but also some limitations.

This first explorative implementation employed a randomized pre-post design with two different intervention groups. However, this approach does not meet the gold standard of randomized control trials in intervention research (e.g., Lupşa et al., 2020; Goldberg et al., 2021). With regards to the sample size and the pandemic situation we did not include a control group without a resilience intervention. However, the absence of a control group means that reported increases in post-test scores might be caused by factors beyond the intervention itself. Additionally, a relatively short period of five days was chosen for this first implementation of the *Resilience Journal*. This short period may have been too brief to detect meaningful changes and effect neuronal plasticity of the students. The long-term effects of the intervention cannot be predicted in the absence of any follow-up measurement and the short intervention period.

Despite these limitations, the inclusion of a journaling method is seen as a strength of this study. Compared to other resilience interventions, this method could be used flexible and cost-efficient during the COVID-19 pandemic. The journaling format was suitable to reach many students without the need for face-to-face meetings or professional trainers and could be delivered daily. The short daily format and the anonymity in journaling interventions could decrease thresholds for participating and we see high practical potential in the journaling method. However, in contrast to most face-to-face interventions the journaling method is an individual task and does not directly provide important interaction or social support.

Additionally, the journaling method was suitable to address daily dynamics in resilience. The development of a framework that conceptualizes resilience as a dynamic process is one of the study’s strengths, especially in training and intervention contexts (e.g., Linz et al., 2020). This dynamic account of resilience grounds the study theoretically and informs the development of the journaling tasks and the daily resilience measure. To the best of our knowledge, MARS is the first published resilience measure to be developed for daily use. However, this new scale is also a limitation; despite encouraging signs, the results of this first implementation must be treated with caution, as the instrument has not yet been validated in a large sample.

Nevertheless, following the recommendations of Joyce et al. (2018), the use of multiple resilience scales supports comparison with other resilience interventions. Another strength of this study is the inclusion of a validated measure of life satisfaction to evaluate the intervention’s effects on well-being (Diener et al., 1985). The non-significant SWLS results refer only to one aspect of subjective well-being and cannot be generalized to well-being as a whole; in other words, the intervention may impact differently on other aspects of well-being. While the selected measures of resilience and life satisfaction all exhibit good internal consistency, the Big Five personality traits returned relatively low values of Cronbach’s alpha. This can be attributed to the small number of items per subscale, and re-test correlations would be more suitable for assessing the reliability of this scale (Rammstedt et al., 2013). The re-test correlations were acceptable, indicating reliable measurement of all variables.

The study was conducted during the COVID-19 pandemic in 2020, and this can be seen as both a strength and a limitation. On one hand, as Joyce et al. (2018) argued, an accurate measure of resilience depends on the presence of a significant challenge or threat, which is not the case in most studies. In the present case, the pandemic fulfilled this requirement, and there was an urgent practical need to develop new online interventions to support university students as a vulnerable population. On the other hand, it is difficult to generalize these insights to other contexts beyond the pandemic. The pandemic could have created additional stress or uncertainty which could have influenced the results. In addition, August is the time in the academic year when students prepare for exams, and the findings might differ at another time of year.

The generalizability of these findings is also limited by the sample, which represents only students from one study program at one German university. While the gender distribution was representative of the study program, the larger proportion of women prohibits generalization to other academic and non-academic populations.

### Implications

This study explored a new approach to online resilience interventions, and the findings have a number of implications for theoretical frameworks and the practicalities of fostering resilience in university settings. To address resilience as a dynamic process, it was necessary to develop a dynamic resilience measure (MARS). These initial findings show that while MARS aligns with the theoretical assumptions, it achieved only low-to-medium correlations with CD-RISC and BRS, indicating that the underlying construct differs from those measured by the other resilience scales. As dynamic definitions of resilience are recommended for the purposes of intervention (Linz et al., 2020), it would be useful to clarify these theoretical differences. In the present case, MARS failed to detect any significant changes in resilience, and any future development of scales measuring short-term changes in resilience must ensure construct and content validity.

Brief resilience scale detected an increase in resilience during the five days of the *Resilience Journal* intervention. While the study’s limitations preclude direct attribution of this effect to the intervention, the findings offer a point of departure for future research on the potential of positive writing, and especially journal-based interventions, as a means of promoting resilience in university students. As there were no significant differences between the two journal versions, there is a need for further research to clarify the underlying mechanisms. In particular, it may prove useful to investigate whether resilience is developed more effectively by a general broadening of attention as proposed by the broaden-an-build-theory (Conway et al., 2012) or by directing attention to resources and abilities. Additionally, future studies should use a control group to eliminate extraneous factors.

The study also has important practical implications for universities. In particular, the challenges of university life in Germany demand appropriate resilience interventions for students. The benefits of online interventions include easy access, reduced inhibition threshold, and flexible use (Kern et al., 2018), making online interventions like the *Resilience Journal* ideal for university use. The ability to reach and support a large population of students in this way makes this a time- and cost-efficient alternative to face-to-face interventions during and beyond the COVID-19 pandemic. The *Resilience Journal’s* flexible format means that it can be used as a standalone tool or to supplement other online and offline resilience interventions. While the *Resilience Journal* was designed for university settings, it could also be used on the same theoretical basis (with relatively minor task changes) by other institutions and organizations.

### Future Research

The present findings confirm the potential of the *Resilience Journal*, which should be further evaluated using other student and non-student samples. The effect sizes reported here serve as a guide for calculating sample sizes. As there were no significant differences between the two journal versions, future research should also investigate similarities and differences in greater depth to clarify the underlying mechanisms activated by the different versions. To that end, future research designs should incorporate control groups of adequate sample size and journal entries should be qualitatively analyzed for further insights.

Additionally, the long-term effects of using the *Resilience Journal* should be explored over a period of several weeks, with follow-up measurement, and recommendations for designing and evaluating resilience and positive psychology interventions should guide future studies (e.g., Joyce et al., 2018; Brewer et al., 2019; Carr et al., 2020; Linz et al., 2020; Goldberg et al., 2021). To generalize the present findings, it will be necessary to replicate the intervention in different faculties, universities, and countries, as well as exploring its use in non-academic contexts. Any new initiatives should be scientifically evaluated–for example, when using the *Resilience Journal* to supplement other resilience interventions or in an offline format.

The new MARS measure introduced here shows promise, but it failed to detect any significant changes. Further validation should involve a larger sample, establishing norm values and capturing day-to-day variations in different settings in the absence of any intervention. By facilitating longitudinal exploration of daily fluctuations in resilience, MARS can help to develop dynamic measures of resilience within individuals and across different time periods. Those insights can then be linked to specific events and traits to provide a better understanding of resilience by building better theoretical models that help to foster resilience and well-being.

## Data Availability Statement

The raw data supporting the conclusions of this article will be made available by the authors, without undue reservation.

## Ethics Statement

Ethical review and approval was not required for the study on human participants in accordance with the local legislation and institutional requirements. The patients/participants provided their written informed consent to participate in this study.

## Author Contributions

ML and CA contributed to the conception and design of the study. ML organized the data collection, performed the statistical analysis, and wrote the first draft of the manuscript. CA supervised the process of data collection and analysis and contributed to the manuscript revision, read, and approved the submitted version. Both authors contributed to the article and approved the submitted version.

## Conflict of Interest

The authors declare that the research was conducted in the absence of any commercial or financial relationships that could be construed as a potential conflict of interest.

## Publisher’s Note

All claims expressed in this article are solely those of the authors and do not necessarily represent those of their affiliated organizations, or those of the publisher, the editors and the reviewers. Any product that may be evaluated in this article, or claim that may be made by its manufacturer, is not guaranteed or endorsed by the publisher.
