# Supplementary material for: The Resilience Journal: Exploring the Potential of Journal Interventions to Promote Resilience in University Students
Source: Front Psychol. 2021 Oct 6;12:702683. doi: 10.3389/fpsyg.2021.702683 (PMC8526970; doi:10.3389/fpsyg.2021.702683)
Supplement: Supplementary file 1 [file Table_1.pdf]

Supplemental Material 1. Means, standard deviations, and pre-requirement tests.

Variable	Mean & Standard Deviation			Pre-Requirements			
	MV	AV	total	t-test	Levene-test	Skewness	Kurtosis
Gender	1.68 (.47)	1.64 (.49)	1.66 (.48)	.42 (.677)	1.66 (.201)	-.69	-1.56
Age	23.58 (2.47)	23.90 (2.42)	23.74 (2.45)	-.641 (.523)	.10 (.756)	.23	-.217
Semester	5.58 (3.23)	5.78 (3.44)	3.44 (2.06)	-.29 (.771)	.17 (.681)	1.63	3.09
Study Program	1.50 (.51)	1.56 (.50)	1.53 (.50)	-.60 (.552)	.61 (.436)	-.12	-2.03
BRS	3.19 (.76)	3.36 (.60)	3.27 (.68)	-1.25 (.215)	2.85 (.095)	-.47	-.52
CD-RISC	2.89 (.37)	2.82 (.39)	2.86 (.38)	.85 (.400)	.058 (.810)	-.22	.36
SWLS	5.32 (1.11)	5.37 (.85)	5.35 (.98)	-.20 (.840)	.92 (.339)	-.59	-.14
BFI-10 N	2.89 (.89)	2.76 (.88)	2.83 (.89)	.73 (.466)	.02 (.886)	.31	-.50
BFI-10 C	3.67 (.78)	3.61 (.92)	3.64 (.85)	.35 (.726)	1.76 (.188)	-.58	.31
BFI-10 E	3.47 (1.07)	3.51 (1.07)	3.49 (1.07)	-.19 (.852)	.65 (.421)	-.28	-.99
BFI-10 O	3.11 (1.25)	3.17 (1.06)	3.14 (1.15)	-.26 (.796)	3.25 (.075)	-.05	-1.06
BFI-10 A	3.47 (.97)	3.31 (.88)	3.39 (.92)	.87 (.389)	.16 (.687)	-.35	-.36
MARS	70.15 (17.45)	66.07 (18.06)	68.11 (17.78)	1.15 (.253)	.33 (.565)	-1.14	1.58

Note. Standard deviations (Mean & Standard Deviation) and p-values (Pre-Requirements) in parentheses; MV = Mastery Version of the Resilience Journal; AV = Attention Version of Resilience Journal; Total = both conditions combined; BRS = Brief Resilience Scale; CD-RISC = Connor-Davidson Resilience Scale; SWLS = Satisfaction with Life Scale; BFI-10 N = Big Five Inventory 10 – Neuroticism; BFI-10 C = Big Five Inventory 10 – Conscientiousness; BFI-10 E = Big Five Inventory 10 – Extraversion; BFI-10 O = Big Five Inventory 10 – Openness; BFI-10 A = Big Five Inventory 10 – Agreeableness; MARS =Monitoring of Actual Resilience State; Gender: 1 = male, 2 = female; Study Program: 1 = Bachelor; 2 = Master.
